# Supplementary material for: First Data on PAE Levels in Surface Water in Lakes of the Eastern Coast of Baikal
Source: Int J Environ Res Public Health. 2023 Jan 9;20(2):1173. doi: 10.3390/ijerph20021173 (PMC9859432; doi:10.3390/ijerph20021173)
Supplement: Supplementary file 1 [file ijerph-20-01173-s001.zip › ijerph-2104227-supplementary.pdf]

Supplementary data

Table S1. Chemicals and materials used in the experimental section

Name	Purity	Source
dichloromethane (DCM)	pesticide grade	Fisher (USA)
n-hexane	pesticide grade	Macron Fine Chemicals (USA)
ethyl acetate	pesticide grade	Macron Fine Chemicals (USA)
methanol	HPLC grade	J.T. Baker (Netherlands)
acetone	HPLC grade	Merk (Germany)
anhydrous sodium sulfate	analytical grade	Merk (Germany)
Glass fiber filters	0.47 µm /47 mm /	Merk (Germany)
SPE tube	Supelclean ENVI-18	Supelco (USA)

Table S2. Additional information on SPE-GC-MS conditions for phthalates

Phthalates			
Parameter	Condition/setting values		
Instrument	GC 7890B MS 7000C (Agilent Technologies, USA)		
Column	HP-5MSUI (30 m × 0.25 mm × 0.25 µm; Agilent Technologies)		
Injection	Split mode, injection volume 1 µL, injection port temperature 280 °C		
Carrier gas	Helium, flow rate 1 mL min–1		
Oven temperature program	60 °C (hold 1 min), to 220 °C (20 °C/min, hold 1 min), to 280 °C (5 °C/min, hold 2 min)		
Interface temperature	280 °C	Ion source temperature	230 °C
Ionization mode	EI, electron energy 70 eV	Monitoring mode	SIM
Compound	m/z	Surrogate	m/z
DMP	**163** (163, 77, 194, 92)	DMP-d4	**167** (81)
DEP	**149** (149, 177, 76)		
DnBP	**149** (149, 223, 104, 76, 205)		
BzBP	**149** (149, 91, 206)	DEHP-d4	**153** (71)
DEHP	**149** (149, 167, 57, 279)		
DnOP	**149** (149, 279)		
Notes: Bold numbers of m/z indicate quantification ions, numbers in parentheses indicate confirmation ions.			

Table S3 The linearity range of PAEs by SPE-GC/MS method

PAEs	Concentration range (µg/L) (6)	Linearity curve	R2	Rt, min	Coefficient of variation (CVm, %)
DMP	1-1000	y=1401.87x−2053.74	0.999	7.69	0.66
DEP	1-1000	y=1695.04x−6956.37	0.999	8.58	0.42
DBP	1-1000	y=2039.90x+497.12	0.999	10.98	1.64
BBP	1-1000	y=549.30x+1173.28	0.999	14.95	0.79
DEHP	1-1000	y=925.46x+7118.06	0.998	17.28	1.63
DnOP	1-1000	y=3220.65x−25825.89	0.998	19.65	2.52
DMP-d4	1-1000	y=1232.39x−12234.52	0.999	7,70	0.61
DEHP-d4	1-1000	y=614.06x−4693.99	0.998	17,29	1.44

Table S4 Accuracy, recovery, limit of detections (MDL), and method quantitation limit (MQL) of PAEs by SPE-GC/MS method

Phthalates	Procedural blanks (ng/L, mean, n=9)	Accuracy (RSD, %)	Recovery range (%)	Surface waters of the lakes	
				MDL (ng/L)	MQL (ng/L)
DMP	5.04	3.00	90.01 ~ 112.12	0.10	0.30
DEP	6.07	1.70	89.05 ~ 110.55	0.10	0.30
DBP	15.12	5.4	100.14 ~ 118.15	0.10	0.30
BBP	9.51	3.47	78.20~101.33	0.30	0.90
DEHP	18.57	4.37	99.85 ~ 115.76	0.41	1.3
DnOP	3.21	6.12	83.11 ~ 105.51	0.70	2.1

International Journal of Environmental Research and Public Health

MDPI

Table S5 **Additional information about geographical and hydrological indicators of the studied lakes**

Characteristics	Lake				
	Arangatui	Bormashevoe	Dukhovoe	Kotokel	Shchuchye
Coordinates	53°33′47″N 109°4′19″E	53°27′39″N 109°0′26″E	53°17′11″N 108°51′44″E	52°50′58″N 108°7′37″E	51°24′58″N 106°32′16,3″E
Area, km²	54.2	1.3	5.84	62.9	4.43
Depth, m	2	2.2	3.7	Mean 4-6, Maximum 14	23
Water supply	the Malyi Chivyrkui River and the Burtui brook, groundwater and fracture-vein water	Fracture-vein and groundwater, atmospheric precipitation	Atmospheric precipitation, groundwater, and fracture-vein water	Atmospheric precipitation, groundwater, brooks, springs from the Ulan-Burgasy ridge, fracture-vein water	Small mountain brooks flowing down from the Khamar Daban ridge, underground springs
Water runoff	This lake is connected to Lake Baikal (Chivyrkuisky Bay) by a channel.	A terminal lake, the level of which is 0.5 m below the level of Lake Baikal.	The Dukhovaya River flows out, and flows into Lake Baikal after 2.5 km	The Istok channel flows out, which through the Kotochik-Turka river system flows into Lake Baikal (15 km after)	Terminal lake
Mineralization, mg/L	70-110	1360-2400	85-115	40-85	220-250
Water class	Freshwater	Saline (soda type)	Freshwater	Freshwater	Freshwater
pH	7.5	8.7-9.0	7.0-9.7	6.8-9.5	7.8-8.2
Water type	Hydrocarbonate-calcium	Hydrocarbonate-sodium	Hydrocarbonate-calcium	Hydrocarbonate-calcium	Hydrocarbonate-calcium
Degree of anthropogenic load	Low*	Low	Medium	High*	High
Anthropogenic impact	Located in a protected area (the Zabaikalsky National Park) in a hard-to-reach swampy area. Fishing.	Located in a protected area (the Zabaikalsky National Park). Visiting tourists with permits	It is part of the recreational area with a ban on economic activity. Amateur fishing. The Barguzin Road runs along the northwest shore of the lake (for 4.7 km).	It is used as a recreational water body with about 40 camping sites and guest houses, located near the Kotokel, Cheremushka, Yartsy and Istok settlements (with total	Used as a recreational water body with more than 20 camping sites and resorts, four children's health camps and five training bases. Fishing.

	population 400–450 people). Fishing.	The Gusinoozersky industrial hub (GRES—a thermal power plant; coal mine) is situated 13 km south of the lake.

International Journal of *Environmental Research and Public Health*

MDPI

Table S6. Physicochemical parameters of surface water in Lakes Arangatui, Bormashevoe, Dukhovoe, Kotokel and Shchuchye. Numerator shows minimum and maximum values, denominator—average.

Parameter	**Arangatui**	**Bormashevoe**	**Dukhovoe**	**Kotokel**	**Shchuchye**
Salinity, mg/L	53.18-78.74	1007.85-1428.29	116.74-126.47	58.15-66.47	287.33-297.71
	71.69	1284.06	120.16	62.55	292.25
Turbidity, NTU	3.80-7.06	17.30-32.80	2.19-3.50	1.56-2.22	0.80-1.31
	5.75	22.53	2.99	1.83	0.99
pH	6.07-6.18	6.83-7.84	6.59-6.86	6.49-6.77	7.60-7.80
	6.15	7.46	6.69	6.61	7.71
DO, mg/L	0.89-6.31	0.12-7.18	0.10-8.66	10.79-13.82	10.82-11.19
	2.74	4.03	5.69	12.11	11.00
PI, mg/L	6.80-10.00	41.60-51.20	16.00-23.20	4.56-6.24	3.84-4.40
	8.60	45.87	20.53	5.52	4.08
COD, mg/L	12.09-16.82	148.56-173.60	45.53-64.72	17.37-33.20	12.75-15.27
	14.56	162.11	61.04	24.17	13.84
NO_2^-, mgN/L	0.007-0.030	0.016-0.025	0.003-0.006	0.001-0.004	0.001-0.002
	0.014	0.019	0.005	0.002	0.001
NO_3^-, mgN/L	0.08-0.13	0.11-0.16	0.10-0.14	0.07-0.12	0.04-0.06
	0.10	0.14	0.11	0.09	0.05
NH_4^+, mgN/L	0.061-0.090	12.572-15.428	0.159-0.221	0.007-0.052	0.003-0.012
	0.076	13.791	0.195	0.033	0.005
PO_4^{3-}, mgP/L	0.110-0.152	0.264-1.418	0.002-0.013	0.006-0.016	0.001-0.002
	0.128	1.005	0.008	0.010	0.001
TP, mg/L	0.12-0.15	1.01-1.84	0.04-0.06	0.03-0.04	0.01-0.02
	0.14	1.49	0.04	0.03	0.02

Table S7. Spearman correlation coefficients between PAE concentrations and water quality parameters

	Turbidity	pH	DO	Salinity	NO_2^-	NO_3^-	NH_4^+	PO_4^{3-}	TP	PI	COD
DMP	0.34	-0.15	-0.16	-0.15	0.34	0.26	0.56**	0.03	0.03	0.51*	0.50*
DEP	0.24	-0.29	-0.15	-0.30	0.19	0.27	0.24	0.25	0.19	0.24	0.08
DBP	0.15	-0.26	0.05	-0.34	0.17	0.36	0.28	-0.10	-0.22	0.26	0.43
BBP	-0.58**	0.35	0,24	0,27	-0.58**	-0.51*	-0.55*	-0.59**	-0.60**	-0.61**	-0.41
DEHP	0.50*	0.05	-0.38	0.23	0.47*	0.18	0.41	0.58**	0.52*	0.39	0.01
DnOP	-0.18	-0.01	0.38	-0.20	-0.24	-0.14	-0.30	-0.01	-0.14	-0.18	0.08
∑PAE	0.38	-0.24	-0.14	-0.20	0.36	0.37	0.40	0.21	0.11	0.40	0.37

* Correlation is significant at the 0.05 level

** Correlation is significant at the 0.01 level
